# How do parents approach nighttime infant care? A grounded theory

**DOI:** 10.3389/frsle.2026.1669946

**Published:** 2026-01-29

**Authors:** Caryn Dooner, Christine Ou, Hana Kim, Lenora Marcellus, Michaela Henry-Dansereau, Jessy Sidhu

**Affiliations:** 1 School of Medicine, Queen's-Lakeridge Health MD FM Program, Oshawa, ON, Canada; 2 Faculty of Human and Social Development, University of Victoria, Victoria, BC, Canada

**Keywords:** 2SLGBTQIA+, care division, couples, dyadic processes, infant sleep, nighttime care, parent sleep, parenting

## Abstract

**Introduction:**

How parent dyads organize and share nighttime caregiving, particularly in the context of gender-roles and diverse family structures, has been given little attention. The aim of this study was to develop a grounded theory explaining how parent dyads manage nighttime infant care, focusing on caregiving practices, decision-making, and their effects on parental roles and well-being.

**Methods:**

Parent dyads with children under 2 years of age who completed an online questionnaire were invited to participate in virtual semi-structured interviews about their nighttime caregiving approaches. Interview transcripts were analyzed using constructive grounded theory methods.

**Results:**

Twenty cisgender heterosexual and 10 2SLGBTQ+ dyads were interviewed. The core category *navigating priorities* was identified, which highlighted the tension created by competing demands and priorities. Parents responded by either *staying the course*—maintaining their current approach—or *changing lanes*—adopting a new strategy—within their unique *dyadic context*. This context encompassed the characteristics and evolving experiences each person brought to their family. This iterative process was triggered whenever tension arose from competing values or priorities.

**Discussion:**

Dyadic context shapes how couples navigate nighttime care priorities. Flexibility in decision-making and active engagement from both partners in adapting to evolving needs promoted mutually supportive nighttime caregiving for families. These findings enhance understanding of shared parenting dynamics in diverse family structures, informing strategies to support parental and infant sleep and well-being.

## 1 Introduction

While infant sleep has been widely studied, the process of nighttime parenting—how parents divide care, make decisions, and adapt routines—has received far less attention (Baglioni et al., 2020; Sadeh et al., 2010; Tikotzky, 2017). Most research to date has focused on mothers, with minimal examination of fathers'/partners' roles in relation to cognitions about infant sleep, nighttime emotional availability, and soothing behaviors (Baglioni et al., 2020). Despite its inevitable impact on sleep and mental health, little is known about how parents collectively organize and share nighttime care. This is a particularly important area of study because inadequate parent and infant sleep is a significant source of stress and a primary concern prompting parents to seek professional help during infancy (Sadeh et al., 2010, 2011).

Despite increasing societal expectations for fathers and partners to be more involved in parenting, women and birthing persons continue to shoulder the majority of childcare, especially at night (Insana et al., 2014; McBean and Montgomery-Downs, 2015; Ou et al., 2022b). Time-use surveys show that mothers perform the majority of caregiving (Callaghan et al., 2024; Statistics Canada, 2017). In both the United States and Canada, mothers spend nearly twice as much time on childcare as their partners (Statistics Canada, 2017; Yavorsky et al., 2015). Nighttime parenting, especially in later infancy, presents a unique opportunity for fathers and non-birthing parents to engage meaningfully with their infants while supporting rest for the birthing parent. By 6 months, many infants sleep for longer stretches at night and get more of their calories from daytime solid foods (Bathory and Tomopoulos, 2017; Henderson et al., 2010). For breastfed infants especially, this reduces the need for night feeds and creates more opportunity for the non-breastfeeding parent to take on nighttime care (Brown and Harries, 2015). This developmental transition may present an opportunity for shared caregiving. Greater paternal/partner parent involvement in childcare—particularly at night—can enhance birthing parent and infant sleep, reduce parenting distress, and improve overall family well-being (Tikotzky et al., 2010, 2015). Yet, fathers and partners remain largely overlooked in research on nighttime infant care, leaving gaps in understanding how their participation influences sleep, mental health, and co-parenting quality.

Inclusive research that captures the experiences of families with 2SLGBTQ+ parents remains limited. The 2SLGBTQ+ community may include but is not limited to Two-Spirit, Lesbian, Gay, Bisexual, Trans, Queer, and a variety of other sexual and gender identities. Although research on nighttime caregiving within 2SLGBTQ+ families is limited, some evidence suggests that these families may adopt more egalitarian approaches to infant care in general (Geay et al., 2020; Goldberg et al., 2012). Studies highlight that infant sleep problems are a source of distress across all family structures, underscoring the need for more inclusive research on family sleep ecology (Alang and Fomotar, 2015; Lévesque et al., 2020). Understanding how diverse families approach nighttime care—including how roles are negotiated, shared, or adapted—can provide valuable insights into supporting parent-infant sleep, mental health, and co-parenting. The purpose of this constructivist grounded theory study was to explore how two-parent families approach nighttime care for infants and young children.

## 2 Methods

### 2.1 Design

As part of a larger mixed-methods study, parent dyads with infants aged 6–24 months who completed an online questionnaire on nighttime caregiving and family sleep were invited to participate in semi-structured interviews about their caregiving approaches. Interview data were analyzed using constructivist grounded theory methods. The study received approval from the institutional ethics review board (22-0337), and informed consent was obtained from all participants prior to the interviews.

### 2.2 Recruitment

Parent dyads were recruited using social media (Facebook, Instagram) and email lists to complete an online survey then invited to participate in a follow-up interview. Eligibility criteria included being a biological or adoptive parent, having a singleton child 2 years of age or under during the study period, fluency in English, and residency in Canada. Families with medically complex children were excluded (e.g., those born preterm or with serious neurological or cardiac conditions). Our recruitment target was 30 dyads, aligning with sample size recommendations for grounded theory (Thomson, 2010). We purposively oversampled 2SLGBTQ+ families to enable meaningful comparative insights across diverse family structures. Of the 86 couples who indicated interest in the follow-up interview via the survey, 40 responded to email correspondence, with 31 scheduling and completing the interview.

### 2.3 Data collection procedures

Semi-structured interviews with parent dyads were conducted via Microsoft Teams and recorded and lasted between 45 and 90 min. Recordings were transcribed verbatim for analysis. Interview questions and prompts evolved based on insights from earlier interviews.

### 2.4 Data analysis

Constructivist grounded theory methods were used to analyze the data (Charmaz, 2006; Chun Tie et al., 2019). Team members listened to audio recordings and completed a read-through of each transcript. The team member responsible for the interview performed initial coding. Coding included first level initial and second level focused coding. All team members had access to each other's coded transcripts and contributed to development of a shared master code list, created through discussion of emerging codes and common patterns across transcripts. Focused coding (organizing initial codes into higher level codes) was completed by first author CD. Focused codes were then developed into categories in consultation with CO and LM. The theory was collaboratively developed by the team.

### 2.5 Rigor

Coding and analysis commenced immediately after each interview, allowing for iterative constant comparison. HK reviewed each transcript for accuracy using the audiovisual recordings and de-identified the data before coding. The verified transcripts were uploaded to MAXQDA for data management and coding. MAXQDA's code bank feature with team-wide access enhanced coding consistency. Within 24 h of each interview, team members wrote interview memos. Analytical memos were written after initial coding of each interview in MAXQDA. Interview memos were shared and accessible to all team members. Analytical memos were reviewed collectively before transitioning from initial to focused coding, and were written during the analysis process. The team engaged in iterative diagramming and consultation to facilitate theory development.

## 3 Results

### 3.1 Participant characteristics

A total of 30 parent-dyads were interviewed; 20 cis-gender heterosexual couples, and 10 couples where at least one person identified as a member of the 2SLGBTQ+ community (Table 1). Infants were an average of 12.8 months of old (range of 7–21 months) at the time of interview. Almost half of the participants identified that their baby's sleep was ‘not a problem at all' (44.3%), while 10 participants (13.1%) identified that their baby's sleep was either a moderate or a serious (3.3%) problem. Other responses included identifying their baby's sleep as ‘a very small problem' (14.8%), ‘a small problem' (23%), or chose not to answer (1.6%). *Navigating Priorities* as the Core Category

**Table 1:** Participant characteristics.

**Characteristics**	**Cis-gender heterosexual couples**		**2SLGBTQ**+ **couples**	
	**Birthing parent**	**Non-birthing parent**	**Birthing parent**	**Non-birthing parent**
Parent age (mean years ± SD)	33.4 ± 4.6	34.3 ±5.0	36.3 ± 4.4	34.6 ± 5.5
Baby age (mean months ± SD)	12.8 ± 4.2		10.1 ± 2.8	
Infant sex (female)	45%		70%	
**Number of children**				
1	17 (80%)		9 (90%)	
2	2 (10%)		1 (10%)	
3	2 (10%)			
**Parent gender identity**				
Woman	20 (100%)	–	7 (70%)	3 (30%)
Man	–	20 (100%)	–	5 (50%)
Genderqueer	–	–	3 (30%)	–
Non-binary	–	–	–	2 (20%)
Cisgender	20 (100%)	20 (100%)	–	–
Trans	–	–	1 (10%)	2 (20%)
**Parent sexual orientation**				
Heterosexual	20 (100%)	19 (100%)	2 (20%)	3 (30%)
Bisexual	–	–	2 (20%)	1 (10%)
Queer	–	–	5 (50%)	4 (40%)
Lesbian	–	–	1 (10%)	2 (20%)
**Parent education**				
University courses	–	–	1 (10%)	–
Post-secondary education completed	20 (100%)	19 (100%)	9 (90%)	10 (100%)
**Parent ethnicity/cultural identity (select all that apply)**				
White	18 (90%)	14 (74%)	8 (80%)	8 (80%)
Aboriginal/Indigenous	–	2 (11%)	2 (20%)	1 (10%)
East Asian	2 (10%)	1 (5%)	–	1 (10%)
Other (please specify)	1 (5%)	1 (5%)	2 (30%)	1 (10%)
**Family household income**				
Under $100k		4 (21%)		4 (40%)
$100–$200k		10 (53%)		5 (50%)
Over $200k		5 (26%)		1 (10%)
Prefer not to answer		–		–
Current employment (yes)	70%	79%	40%	70%
Parent born in Canada (yes)	90%	84%	100%	70%

**Figure 1:**
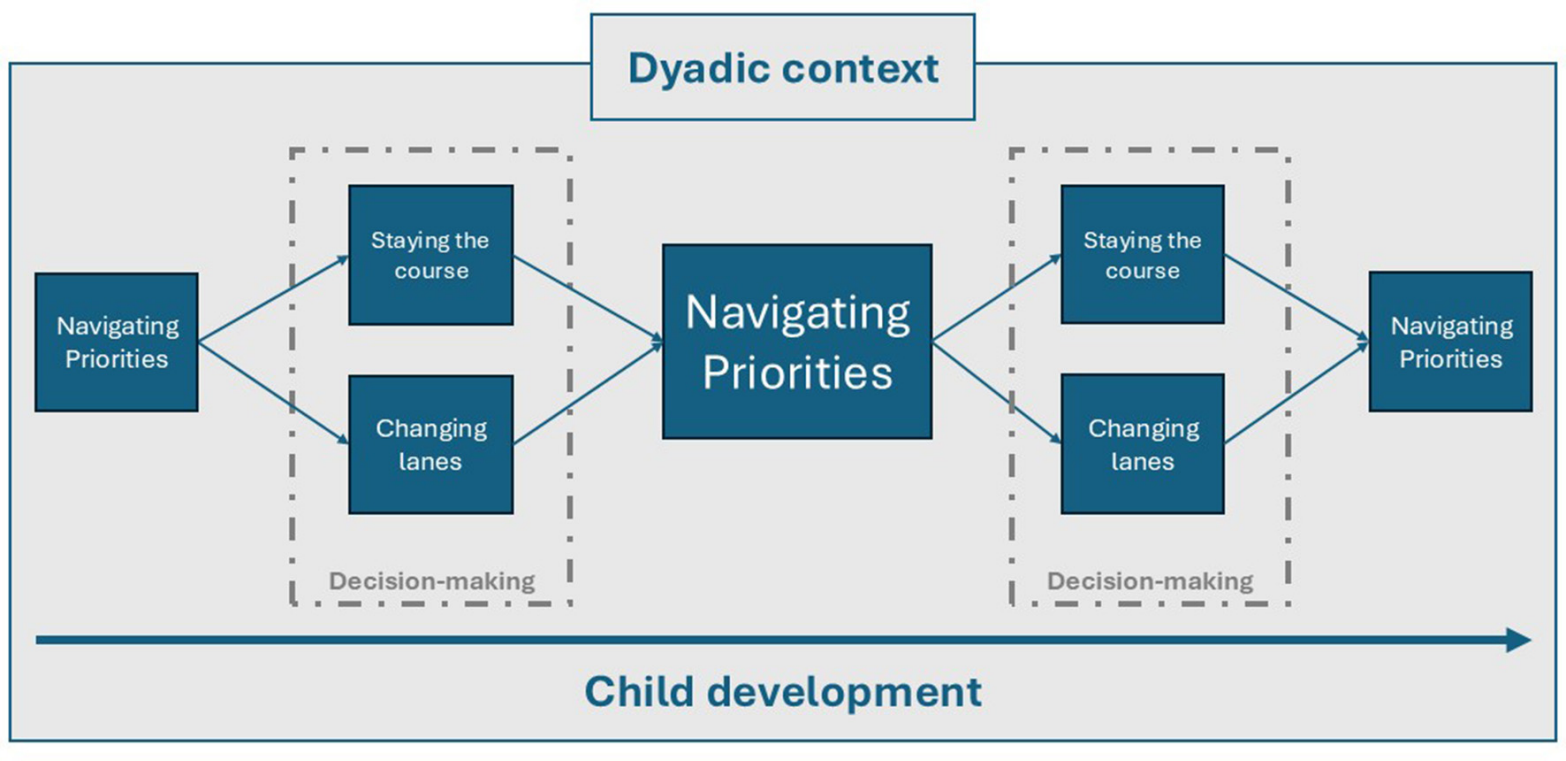
Grounded theory model of how co-workers divide nighttime care of their infants.

From participants' accounts of their approach to organizing nighttime infant care, the core category of *navigating priorities* emerged (Figure 1). *Navigating priorities* referred to the convergence or conflict of tasks, values, and responsibilities requiring parents to decide on a course of action. To manage these tensions, participants either chose to *stay the course*—maintaining existing strategies—or *change lanes* by adopting new strategies. The decision-making process of *navigating priorities* recurred over time as infants grew through different developmental stages and as parent/family circumstances evolved. *Navigating priorities, staying the course*, and *changing lanes* operated within the *dyadic context*—the evolving characteristics, circumstances, and experiences that each parent brought to the parenting partnership. These core components—navigating priorities, staying the course, and changing lanes are mapped in Figure 1 and serve as the organizing structure for this section. In Table 2, participant quotes are shown with age, gender, and sexual orientation characteristics.

**Table 2:** Dyad quotes and characteristics.

**Dyad**	**Birthing parent**	**Partner**	**Quotes**
**2SLGBTQ**+			
Dyad 1	Genderqueer person; queer Age: 38	Cisgender woman; queer Age: 38	“*We have to make it a priority; if we are going to do something, we have to put it in our app and we stick to the plan. If we don't do that, we drop it at the end of the day.”(Birthing parent)*
Dyad 2	Genderqueer person; lesbian Age: 41	Cisgender woman; lesbian Age: 37	“*We intended to try to keep things as similar as possible, but couldn't really do that*. (*Partner) “It evolved kind of by necessity. I can't be there for our toddler's nighttime routine because I'm feeding.” (birthing parent) “[We are] team-oriented.” (Birthing parent)*
Dyad 3	Cisgender woman; queer Age: 35	Non-binary person; queer Age: 36	“*Being queer parents [and] having a different family structure, we've already done a lot of work at deconstructing gendered roles. And that has made a big difference in how we feel about dividing the labor around sleep and making it equitable. When we are talking about how we handle sleep and the conversations we've had and how we've planned everything out, I think that [communicating] plays a really important role.” (Birthing parent)*
Dyad 4	Cisgender woman; lesbian Age: 39	Cisgender woman; queer Age: 26	“*Three days later [baby] was getting good at putting [themselves] to sleep.” (Partner)*
Dyad 5	Cis-gender woman; heterosexual Age: 30	Cisgender man; bisexual Age: 33	“*The baby is just more comfortable with her mom for upsetting experiences. I'm her play friend, and mom is her cuddle friend… she just fights me until mom shows up.” (Partner)*
Dyad 6	Cisgender woman; queer Age: 32	Non-binary person; queer Age: 27	“*She just wasn't a great sleeper until we did sleep training.” (Birthing parent) “You really do need to set up those boundaries and set up those expectations.” (Partner)*
Dyad 7	Cisgender woman; bisexual Age: 37	Cis-gender man; heterosexual Age: 39	“*[I'm a] high sleep needs.” (Partner) “I really wanted to protect his mental health and sleep.” (Birthing parent)*
**Cis-gender heterosexual**			
Dyad 1	Woman Age: 25	Man Age: 30	“*We don't.” (Birthing parent and partner)*
Dyad 2	Woman Age: 37	Man Age: 37	“*This is survival mode. I used to read one or two novels a month. It's something I've had to give up.” (Birthing parent) “His sleeping schedule starts at 7:00 PM. Weekdays or weekends, it's the same. After he gets breastfed, I take over and try to make him sleep.” (Partner) “What most moms seem to be doing here, is sleep training, and hardcore sleep training…so put the baby down at, you know, 7:30 or 8 and they're not allowed out of their crib until 7:00 o'clock the next morning, no diaper changes, no feeds… They just get ignored, which I think is really awful.” (Birthing parent) “[Baby] is happier sleeping in the same bed with us and staying in the same room seems better for him.” (Birthing parent)*
Dyad 3	Woman Age: 37	Man 34	“*She would just escalate and get upset. The second [Mom] would come in, she'd stop. It was not fair.” (Partner) “[Baby] barely cries when we're together, because I have a boob.” (Birthing parent) “It's really tricky because [first daughter] was in the bed, so the baby can't be loud. I just have to hold the boundary and I haven't been able to properly do so.” (Birthing parent) “[Baby] co-sleeps on [Mom]'s side and I sleep kind of deeply anyway. [Mom] fed [baby] at night. Just no point in me doing it.” (Partner) “My dad was helpful. He would come and take the baby away so I could get naps. Sleep when you can.” (Birthing parent)*
Dyad 4	Woman Age: 43	Man Age: 48	“*I'm too tired to do anything anyway.” (Partner) “There's always this thing that I meant to do and I didn't have the time.” (Birthing parent) “If we do deviate from the routine a lot, it has consequences. We went to my mother's for lunch and were leaving later than I would have wanted. On the way home he was asleep for probably about 40 minutes, and he didn't go to bed until late that night.” (Birthing parent) “I feel so old going to bed at 10pm…If I'm losing my patience, or if my needs aren't being met, then his needs are not being met as well as they could be either. Before he was born, I don't think I'd been in bed before midnight since I was in my teens.” (Partner)*
Dyad 5	Woman Age: 39	Man Age: 36	“*Once we moved into our house, everything was different—[Mom] would go to a different room and get some sleep.” (Partner) “…a completely different environment, and [baby] would be sharing a room with us.” “[We were] so desperate for sleep” (Birthing parent) “My mom would come in because she's the early riser anyway, and she would swoop in like an angel and steal [baby] away in the dark of night.” (Birthing parent)*
Dyad 6	Woman Age: 31	Man Age: 29	“*It's 50-50 really…” (Partner)*
Dyad 7	Woman Age: 36	Man Age: X^*^	“*[It's an] equal split” (Birthing parent) “We didn't worry so much because we know we could keep a kid alive and we know that they'll grow out of it.” (Partner) ”You know you're useless because you can't breastfeed” (Partner)*
Dyad 8	Woman Age: 29	Man Age: 29	“*Yeah, the continual crying. I don't know. It just rattles me.” (Birthing parent) “I was more willing to go down and soothe [baby].” (Partner) “[Baby's] trying phase of wanting to cry for an hour or two before bed won't last forever.” (Partner) “It's ok to just put the baby down, step away for a couple minutes.” (Birthing parent)*
Dyad 9	Woman Age: 31	Man Age: 25	“*There was a point in our son's life where we would have to bounce him on an exercise ball for 20 minutes and then hope … he would sleep.” (Birthing parent) “Sleep training is definitely a controversial topic. We used a sleep training method called Ferber…I would say [Dad] exclusively had the responsibility.” (Birthing parent)*
Dyad 10	Woman Age: 31	Man Age: 41	“*I couldn't get into a deep sleep because I was terrified of something happening to him. Like we're increasing the risk of SIDS, I don't want anything to happen.” (Birthing parent) “We chose the option where crying intervals get longer and longer… that method worked for us.” (Partner)*

X^*^ = missing data.

### 3.2 Navigating priorities

The core category of navigating priorities captures the ongoing, cyclical process situated in the dyadic context, through which parents negotiate their values and present circumstances. This process began with recognizing and making sense of the competing priorities they were managing—an essential precursor to navigating them effectively. Competing priorities often gave rise to tension, reflecting the conflicting demands that parents faced. Many parents expressed feeling pressed for time and overwhelmed when balancing their children's needs with their own. Priorities included activities of daily living (e.g., food preparation, laundry), hygiene, infant care (e.g., feeding, sleeping), paid employment, and making time for both one's partner and oneself. Navigating priorities is revisited whenever competing demands arise, requiring parents to choose between staying the course or changing lanes.

In response to competing demands, parents often prioritized infant care above their own needs. Striking examples of how parents navigated the balance between personal time and parenting demands emerged when each dyad was asked, “How do you make time for yourselves?” Responses varied widely from a point-blank “We don't”, to a minority of dyads intentionally setting aside time for activities together. One cis-gender heterosexual mother expressed the sacrifices involved, and shared, “This is survival mode. I used to read one or two novels a month. It's something I've had to give up.” A genderqueer identifying birthing parent, shared “We have to make it a priority; if we are going to do something, we have to put it in our app and we stick to the plan. If we don't do that, we drop it at the end of the day.” This range of responses illustrates how parent dyads adapted differently to the pressures of early parenthood, often making personal sacrifices to prioritize family needs.

In navigating nighttime care, parent dyads juggled differing priorities and perspectives while adapting to their circumstances and their child's needs. Several parents noted breastfeeding as a priority but that it hindered shared nighttime care involvement. One father from a cis-gender heterosexual-identifying dyad described how the baby's preference for the mother at bedtime created challenges: “She would just escalate and get upset. The second [Mom] would come in, she'd stop. It was not fair.” The mother agreed: “[Baby] barely cries when we're together, because I have a boob.” Other dyads prioritized setting loving limits to maintain control over nighttime care. One cis-gender heterosexual father shared, “His sleeping schedule starts at 7:00 p.m. Weekdays or weekends, it's the same. After he gets breastfed, I take over and try to make him sleep.” Despite the mother's preference not to sleep train, the father made efforts to set consistent routines. Priorities were also shaped by shifting household demands. A lesbian couple described how the arrival of a second child disrupted their previously shared routine of alternating bedtime duties: “We intended to try to keep things as similar as possible, but couldn't really do that. Her partner, the birthing parent, added, “It evolved kind of by necessity. I can't be there for our toddler's nighttime routine because I'm feeding.” Priorities dyads navigated, including bedtime routines and nighttime care, were shaped by their unique dyadic context. The dyadic context evolved continuously over time, concurrently with childhood developmental milestones such as teething and increasingly independent motor activity.

Fatigue and limited time were significant factors affecting how parents navigated their priorities at night. Even when brief windows for personal time emerged, such as the time between baby and parent bedtime, fatigue often prevented parents from taking advantage of these opportunities. One father from a cis-gender heterosexual-identifying dyad shared: “I'm too tired to do anything anyway.” The mother from the same dyad added, “there's always this thing that I meant to do and I didn't have the time.” Fatigue and time constraints played a key role in shaping how evening and nighttime routines were carried out.

### 3.3 Dyadic context

In the model, *dyadic context* reflected the unique realities of each dyad and provided the confines within which *navigating priorities* and its subcategories, *staying the course* and *changing lanes*, operated. Parents held both individual and shared ideas, beliefs, values, and goals for parenting while some also navigated additional complexities: alternate housing arrangements, pre-existing health conditions, employment constraints, or blended family structures. Parenting goals were shaped by socio-environmental and cultural contexts, influencing how dyads divided and organized nighttime caregiving responsibilities. A cis-gender heterosexual-identifying dyad described how the physical space in their in-laws' home where they previously lived had posed a challenge: the father stated, “Once we moved into our house, everything was different—[Mom] would go to a different room and get some sleep.” The mother added that at their in-laws', they had “a completely different environment, and [baby] would be sharing a room with us.” She noted they were “so desperate for sleep” due to frequent night wakes while sharing a room.

*Dyadic context*, as a blend of personal history, present circumstances, and sociocultural influences was illustrated by a 2SLGBTQ+ birthing parent who identified as queer partnered with a non-binary queer co-parent. They shared:

*Being queer parents [and] having a different family structure, we've already done a lot of work at deconstructing gendered roles. And that has made a big difference in how we feel about dividing the labour around sleep and making it equitable. When we are talking about how we handle sleep and the conversations we've had and how we've planned everything out, I think that [communicating] plays a really important role*.

This couple's experience underscored how the *dyadic context*, including each partner's values and shared identity as queer parents, shaped their nighttime parenting approach. By critically reflecting on societal norms around one parent being a primary caregiver, this couple was able to prioritize fairness and shared responsibility. A different dyad comprised of a 2SLGBTQ+ genderqueer lesbian and a cisgender lesbian woman described their dyad as “team-oriented” when facilitating their baby's sleep. In contrast, although not universally, many cis-gender heterosexual-identifying parents described mothers as the primary caregiver despite initially claiming “it's 50–50 really” or referring to nighttime care as an “equal split”.

Dyads' attitudes and beliefs about infant sleep emerged as a key contextual factor in organizing nighttime care, ranging from attachment-style parenting and bed sharing to encouraging independent sleep through sleep training. A queer-identifying birthing parent shared how she and her partner decided to sleep train after consulting friends, who described sleep training as life changing. After sleep training, “3 days later [baby] was getting good at putting [themselves] to sleep.” In contrast, a mother from a cis-gender heterosexual-identifying dyad expressed opposition toward sleep training:

*What most moms seem to be doing here, is sleep training, and hardcore sleep training…so put the baby down at, you know, 7:30 or 8 and they're not allowed out of their crib until 7:00 o'clock the next morning, no diaper changes, no feeds… They just get ignored, which I think is really awful*.

As the mother explained their preference for co-sleeping over sleep training, the father agreed that bed-sharing was positive, stating, “[Baby] is happier sleeping in the same bed with us and staying in the same room seems better for him.” Many factors and ideas shaped the dyadic context within which parent couples operated, influencing how they navigated priorities and made decisions about nighttime care. The dyadic context served as the framework for deciding whether to *stay the course* (maintain the status quo) or *change lanes* (adjust their approach) in managing nighttime care. *Dyadic context* captures both enduring aspects of the relationship (such as shared values, personal histories, and social identities) and the couple's ongoing interactions (such as responsiveness, collaboration, or conflict in navigating nighttime care). In this model, dyadic context functions not only as a backdrop but actively shaped how parents perceived and acted on the challenges of infant sleep, providing a key lens through which decision-making processes unfold.

### 3.4 Staying the course

When deciding to *stay the course* or *change lanes, staying the course* involved making minimal or no change to nighttime care. Often this was because changing behaviors, such as sleep training, transitioning from bed sharing to the crib, or asking partners to increase involvement, was seen as impractical or undesirable. One mother from a cis-gender heterosexual-identifying dyad with two children expressed wanting to night wean but felt unable to do so: “It's really tricky because [first daughter] was in the bed, so the baby can't be loud. I just have to hold the boundary and I haven't been able to properly do so.” She was confident night weaning would eventually happen – her priority was to avoid waking the older child. The father explained his limited involvement: “[Baby] co-sleeps on [Mom]'s side and I sleep kind of deeply anyway. [Mom] fed [baby] at night. Just no point in me doing it,” illustrating how breastfeeding can lead to inequitable divisions of labor. As a result, Mom continued to cope with fragmented sleep to tend to the baby at night. Thus, when faced with navigating priorities, some parents chose to stay the course when making a change involved a trade-off they were not prepared to make.

Parents also found themselves entrenched in patterns of behavior that made it difficult to change course. One father, who identified as bisexual, shared, “The baby is just more comfortable with her mom for upsetting experiences. I'm her play friend, and mom is her cuddle friend … she just fights me until mom shows up.” For another cis-gender heterosexual dyad, the mother expressed her preference for her partner to handle nighttime care: “Yeah, the continual crying. I don't know. It just rattles me.” This resulted in the father adapting to the situation: “I was more willing to go down and soothe [baby].” These examples illustrate how situational caregiving patterns and infant preferences can limit flexibility in nighttime care routines.

Other reasons for *staying the course* included prioritizing the consistency of established sleep routines and rationalizing current, albeit suboptimal, practices. One cis-gender heterosexual-identifying mother explained the challenges her family faced when deviating from their routine: “If we do deviate from the routine a lot, it has consequences. We went to my mother's for lunch and were leaving later than I would have wanted. On the way home he was asleep for probably about 40 min, and he didn't go to bed until late that night.” Maintaining consistency in their routine reinforced the need to set boundaries with extended family, helping them manage interactions that could disrupt their child's sleep schedule.

Parents' ability to rationalize why change was not worthwhile was another factor in the decision to *stay the course*. Several parents identified the temporary nature of their circumstances, as one father (part of a cis-gender heterosexual-identifying dyad) summarized: “[Baby's] trying phase of wanting to cry for an hour or two before bed won't last forever.” Mom agreed: “It's ok to just put the baby down, step away for a couple minutes.” The idea that certain parenting challenges were temporary helped some parents accept situation that would otherwise feel untenable. One cis-gender heterosexual-identifying father, explained: “we didn't worry so much because we know we could keep a kid alive and we know that they'll grow out of it.” Focusing on the temporary nature of circumstances helped normalize their demanding routine.

### 3.5 Changing lanes

Nighttime parenting often needed to evolve in response to changing infant needs or parental factors. These changes ranged from minor adjustments, like switching the order of reading and feeding, to larger changes such as implementing sleep training. For example, one queer-identifying mother explained “She just wasn't a great sleeper until we did sleep training.” The same 2SLGBTQ+ dyad identified that in addition to sleep training, limit-setting was key to maximizing sleep: “You really do need to set up those boundaries and set up those expectations.” For this dyad, the act of setting boundaries was a step toward improving family wellbeing.

To *change lanes*, significant planning was often required to facilitate new approaches to nighttime care. Deciding what to change often took research and input from various sources, and the decision reflected the dyad's values while addressing the new challenges. For example, one cis-gender heterosexual-identifying mother stated, “There was a point in our son's life where we would have to bounce him on an exercise ball for 20 min and then hope … he would sleep,” describing the strain on both parents that fueled the need to make changes. After gathering information on infant sleep, the mother decided that sleep training was the best approach to address their sleep challenges and coordinated with the father, who took the lead on implementation. She explained, “Sleep training is definitely a controversial topic. We used a sleep training method called Ferber … I would say [Dad] exclusively had the responsibility.”

Parents also changed their behavior to accommodate their present circumstances. One cis-gender heterosexual-identifying father stated that he is “high sleep needs”. The mother, who identified as a bisexual woman, stated that “I really wanted to protect his mental health and sleep.” This dyad shared that during the early baby stages, while Mom was on leave and Dad had resumed work, their routine involved the mother napping after dinner so the father could rest through the night, rather than both parents having to be awake overnight. A father from a cis-gender heterosexual-identifying dyad stated, “I feel so old going to bed at 10 p.m. … If I'm losing my patience, or if my needs aren't being met, then his needs are not being met as well as they could be either. Before he was born, I don't think I'd been in bed before midnight since I was in my teens.” These reflections highlight how parents *change lanes* by adjusting their routines and sleep habits to fit their children's needs.

*Changing lanes* sometimes involved parents drawing on their support systems. One cis-gender heterosexual-identifying mother struggled with fragmented sleep due to frequent night waking's: “My dad was helpful. He would come and take the baby away so I could get naps. Sleep when you can.” Many parents identified their support systems as a crucial factor in making changes, particularly those aimed at maximizing sleep. Another cis-gender heterosexual mom identified that her mother was crucial in facilitating her and her husband's ability to rest: “My mom would come in because she's the early riser anyway, and she would swoop in like an angel and steal [baby] away in the dark of night.”

Another important driver of change were considerations related to infant safety. Not all dyads expressed concern about infant safety related to sleep, but it was a major driver of change for those who did. One cis-gender heterosexual-identifying dyad was particularly concerned about their practice of co-sleeping with the baby. The mother shared: “I couldn't get into a deep sleep because I was terrified of something happening to him. Like we're increasing the risk of SIDS, I don't want anything to happen.” The father identified that having some support from a doula was key in transitioning away from co-sleeping to facilitate parental sleep: “We chose the option where crying intervals get longer and longer … that method worked for us.” Safety concerns were significant drivers of decisions about a child's nighttime routine and sleep environment.

## 4 Discussion

*Navigating priorities* was identified as the core category, emerging from parents' reflections on their multiple responsibilities and the ongoing decisions required to manage them. Participants could decide to *change lanes* by pursuing a different approach, or to *stay the course* by continuing without making a change. *Changing lanes* often occurred when nighttime care became unsustainable or took a toll on parental mental health. In contrast, *staying the course* can be attributed to change being seen as unnecessary such as in the case of families who described their infants as good sleepers, or when parents were unable to break from the current routine for a variety of reasons. The *dyadic context* influenced *navigating priorities, staying the course*, and *changing lanes*. Navigating priorities were continuously shaped by the evolving characteristics and experiences of each parent. The iterative process of *navigating priorities* within the *dyadic context* unfolded against the backdrop of infant growth, including developmental milestones such as teething and illness. Infant sleep quality also appeared to shape whether dyads stayed the course or changed lanes. For nearly half of participants who described their infant's sleep as unproblematic, there may have been less perceived need for change. In contrast, those who reported more problematic infant sleep may have had greater impetus to adjust their approach to nighttime care.

Participants represented a sample of gender- and sexual identity-diverse parents, including 20 cisgender-cis-gender heterosexual dyads and 10 dyads in which at least one member identified as part of the *2SLGBTQ*+ community, each with a unique *dyadic context*. Dyads who proactively communicated and collaboratively managed nighttime care—especially when one partner took on additional responsibilities while the birthing parent managed demanding tasks like breastfeeding and soothing—generally reported greater satisfaction. This contrasted with dyads that followed traditional gender roles: non-birthing partners often deferred involvement with justifications such as, “you know you're useless because you can't breastfeed” rather than seeking alternative ways to contribute. While some heterosexual dyads described their caregiving arrangements as equal, some accounts revealed persistent gendered divisions—particularly around breastfeeding and emotional labor—suggesting a disconnect between perceived and actual caregiving equity. A recent study found that mothers remain the primary nighttime caregivers even when mothers have returned to work or when no longer breastfeeding—and this unequal distribution is associated with increased maternal distress and lower relationship satisfaction, highlighting the need to promote greater paternal involvement (Breton et al., 2025). In our study, 2SLGBTQ+ participants tended to describe deliberate, reciprocal negotiation of roles, suggesting that caregiving equity may be rather more actively constructed in some of these dyads. These interpretations should be viewed in light of the small, self-selected sample and the possibility that participants with more egalitarian or reflective caregiving dynamics were more inclined to participate.

Dyads in which both partners identified as part of the 2SLGBTQ+ community often described open, reciprocal communication about nighttime care—more so than was evident in many, though not all, straight couples or mixed-orientation dyads. These findings align with research showing that same sex dyads tend to strive for egalitarian division of tasks (Appelgren Engström et al., 2019; Geay et al., 2020; Goldberg et al., 2012). Our research underscores the need to challenge gendered assumptions in relationships, so that more equitable, collaborative approaches to parenting (modeled in many 2SLGBTQ+ families) are adopted in heteronormative relationships. In heteronormative relationships, inequities in caregiving are a known source of distress and anger (Billotte Verhoff et al., 2023; Callaghan et al., 2024; Lockman, 2019) with 22% of women indicating clinically significant levels of anger in the first year after birth (Ou et al., 2025). Studies have found that women report anger and resentment when expected to manage the majority of nighttime infant caregiving and cope with chronic sleep disruption during the transition to parenthood (Ou et al., 2022a,b).

Individuals in a dyad often need to balance priorities such as activities of daily living, infant care, and making time for their partners and themselves. To facilitate change, parents identified using their support systems as helpful. For example, having family nearby can ease childcare demands and allow dyads to prioritize rest, which in turn may give parents more capacity to manage shifting priorities. In our study, participants described distress related to poor sleep, particularly among birthing parents. These narratives echo prior research showing that self-reported poor sleep quality over the perinatal period is linked to lower perceived social support during pregnancy, and that greater support can buffer the impact of sleep disturbances on postpartum depression and stress (Tomfohr et al., 2015; Camisasca et al., 2021). More studies are needed to examine the relationships between social support and postpartum parental sleep quality.

Implications of this study must be understood within the broader societal context in which new parents are navigating the transition to early parenthood. Expectations of joy, bonding, and balance are often replaced by a reality of sleep deprivation, unequal caregiving responsibilities, and limited access to both social and structural supports. While extra-dyadic support is critical to helping parents manage early caregiving demands (Bridgers and Fox, 2024), not all families can rely on these networks. Structural factors, including inflexible or unequal parental leave, shape how caregiving responsibilities are distributed between birthing and non-birthing parents (Doucet and McKay, 2020). When policies do not offer sufficient incentive or limited leave options for non-birthing parents, this reinforces norms that position birthing parents as primary caregivers–an expectation further amplified by intensive parenting ideals (Faircloth, 2021). These constraints directly affect who takes on nighttime care. Additionally, sleep difficulties are often normalized as inevitable and short-lived, reinforcing the notion that intervention is unnecessary (Sadeh et al., 2011). Addressing these socio-structural factors is essential to fostering equitable and sustainable nighttime caregiving practices for parents.

Finally, our findings suggest that dyadic negotiation operates as a relational mechanism through which the broader family context influences infant and caregiver sleep outcomes. Extending the transactional model of sleep (Sadeh et al., 2010; Tikotzky, 2017), we specify how couples navigate nighttime caregiving through ongoing decision-making, shaping how responsibilities are divided and routines sustained or adapted. Future research could examine how perceived fairness and gendered expectations in couples' negotiations shape sleep outcomes, clarifying the role of social dynamics in sleep regulation models.

### 4.1 Strengths and limitations

To our knowledge, this study is the first to examine dyad's (both cis-gender heterosexual and 2SLGBTQ+ dyads) approaches to nighttime infant care by interviewing both partners. This study has limitations that should be considered when interpreting the findings. Our sample is predominantly white, well-educated, and high-income, which may limit the transferability of our findings to more structurally marginalized families. Parenting priorities and dyadic strategies may differ significantly in contexts shaped by financial precarity or single parenthood, or cultural norms around caregiving. For instance, families with fewer material or social supports may have less flexibility to “change lanes”—such as not being able to enlist help from extended family or moving the infant to a separate room when space is limited—and may instead be forced to adapt to constraints. Sleep routines and parental expectations are also shaped by cultural norms. In many cultures, practices such as co-sleeping are common and not viewed as problematic, resulting in different appraisals of infant sleep challenges compared to Western contexts where solitary sleep and infant independence are emphasized (Mindell et al., 2010). We did not have any participants that identified as adoptive parents. Most of our research participants identified that their families sleep was good. Poor sleep would likely prevent a dyad from dedicating time to participating in this study. As such, our proposed model may not fully capture the dynamics of families experiencing clinically significant sleep disruptions, elevated parental distress, or structural barriers such as poverty or housing instability. Importantly, the model reflects caregiving negotiation *processes* rather than sleep outcomes—and should be interpreted within this boundary. Future work is needed to assess how such decision-making processes play out in more constrained contexts or among families facing more severe infant sleep challenges.

Additionally, our study included male partners who were willing to participate in research, which may not fully represent the perspectives of other cis-gender heterosexual dyads. Recruiting fathers for perinatal and parenting research remains a well-documented challenge (Keys et al., 2019). Furthermore, we were unable to recruit male participants in gay relationships, which limits the transferability of our findings to the broader 2SLGBTQ+ community.

### 4.2 Conclusions

Disrupted parental sleep is a well-documented challenge in the first 2 years postpartum. Limited research has examined how parent dyads organize nighttime caregiving and navigate its impact on family wellbeing or sleep. Our findings suggest that parents continuously negotiated competing priorities when organizing nighttime care within their unique and dynamic *dyadic context*. *Changing lanes* could be labor-intensive and required parents to perceive change as feasible and necessary. In contrast, *staying the course* involved enduring the current situation when change was impossible or unnecessary. When supporting parents struggling with infant sleep, it is crucial to understand what factors influence decision-making in response to competing priorities. Ultimately, effective nighttime caregiving was shaped by mutual decision-making, adaptability, and equitable active involvement.

## Data Availability

The raw data supporting the conclusions of this article will be made available by the authors, without undue reservation.
